# Clinical benefit of gluten-free diet in screen-detected older celiac disease patients

**DOI:** 10.1186/1471-230X-11-136

**Published:** 2011-12-16

**Authors:** Anitta Vilppula, Katri Kaukinen, Liisa Luostarinen, Ilkka Krekelä, Heikki Patrikainen, Raisa Valve, Markku Luostarinen, Kaija Laurila, Markku Mäki, Pekka Collin

**Affiliations:** 1 Department of Neurology, Päijät-Häme Central Hospital, Lahti, Finland; 2 Department of Gastroenterology and Alimentary Tract Surgery, Tampere University Hospital, Tampere, Finland; 3 School of Medicine, University of Tampere, Finland; 4 Department Internal Medicine, Päijät-Häme Central Hospital, Lahti, Finland; 5 University of Helsinki, Department of Education and Development in Lahti, Finland; 6 Department of Surgery, Päijät-Häme Central Hospital, Lahti, Finland; 7 Paediatric Research Centre, University of Tampere and Tampere University Hospital, Tampere, Finland

## Abstract

**Background:**

The utility of serologic screening for celiac disease is still debatable. Evidence suggests that the disorder remains undetected even in the older population. It remains obscure whether screening makes good or harm in subjects with long-standing gluten ingestion. We evaluated whether older subjects benefit from active detection and subsequent gluten free dietary treatment of celiac disease.

**Methods:**

Thirty-five biopsy-proven patients aged over 50 years had been detected by serologic mass screening. We examined the disease history, dietary compliance, symptoms, quality of life and bone mineral density at baseline and 1-2 years after the commencement of a gluten-free diet. Symptoms were evaluated by gastrointestinal symptom rating scale and quality of life by psychological general well-being questionnaires. Small bowel biopsy, serology, laboratory parameters assessing malabsorption, and bone mineral density were investigated.

**Results:**

Dietary compliance was good. The patients had initially low mean serum ferritin values indicating subclinical iron deficiency, which was restored by a gluten-free diet. Vitamin B12, vitamin D and erythrocyte folic acid levels increased significantly on diet. Celiac patients had a history of low-energy fractures more often than the background population, and the diet had a beneficial effect on bone mineral density. Alleviation in gastrointestinal symptoms was observed, even though the patients reported no or only subtle symptoms at diagnosis. Quality of life remained unchanged. Of all the cases, two thirds would have been diagnosed even without screening if the family history, fractures or concomitant autoimmune diseases had been taken carefully into account.

**Conclusions:**

Screen-detected patients benefited from a gluten-free diet. We encourage a high index of suspicion and active case-finding in celiac disease as an alternative to mass screening in older patients.

## Background

Evidence suggests that the incidence of celiac disease increases with age [1,2]. Physicians' lack of alertness in the older people may result in a significant delay in diagnosis, as celiac disease is widely deemed to be a condition affecting younger subjects. Indeed, the majority of older celiac disease patients have remained undetected, often due to the absence of symptoms [3,4]. It is reasonable to assume that, due to long gluten exposure, older patients with untreated celiac disease may be disposed to severe nutritional deficiencies, even when they are seemingly asymptomatic. In particular, the rate of bone loss is accelerated in women after the menopause, likewise in men at the same age. We know that even young and asymptomatic patients with celiac disease may have reduced bone mineral density while untreated [5], and the condition might be even more evident in older [6].

On the other hand, a lifelong gluten-free diet is restrictive and may also increase the burden of illness and impairs quality of life [7,8]. Especially in the older subjects the diet may not be well tolerated, as patients will have adopted lifetime dietary habits which may be hard to break. Moreover, if a gluten-free diet does not necessarily produce any clinical improvement, screen-detected cases may not be motivated to adhere strictly to it. Therefore, to clarify the benefit of serologic screening for celiac disease in older population, a follow-up of the previously undiagnosed patients identified by screening is required [6].

In this prospective study we evaluated the benefits of active serologic population-based mass screening for celiac disease and subsequent dietary treatment in subjects over 50 years of age. The aim was to establish whether celiac disease should be rigorously searched for in the older people.

## Methods

### Patients and study design

We identified screen-detected new celiac disease patients enrolled from a cohort representing the older Finnish population. The original study population comprised 4272 randomly selected individuals born in the years 1946-50, 1936-40 and 1926-30, and was defined for a 10-year research project on ageing and well-being (Good Ageing in the Lahti region = GOAL) http://www.palmenia.helsinki.fi/ikihyva/InEnglish.html. In 2002, altogether 2815 subjects (66%) consented to participate and serum samples were drawn and stored at -20°C until used. Anti-tissue transglutaminase antibodies were tested from the stored sera in 2004, and 48 new seropositive cases were identified; their diagnostic work-up has been published elsewhere [2]. Four had started a gluten-free diet before enrolment, 39 of the remainder consented to a small bowel biopsy, and villous atrophy with crypt hyperplasia compatible with celiac disease was found in 35. These 35 comprised the current study group; their median age was 61 years (range 52-76) and 57% were female.

At baseline the patients were interviewed for their family history, associated disorders and symptoms of celiac disease. Nutritional condition and quality of life were evaluated. The patients were advised to start a gluten-free diet, and dietary counseling was carried out by a gastroenterologist and a dietician. To ensure dietary response and adherence to a gluten-free diet, an interview by the dietitian, small bowel biopsies and celiac disease antibodies were investigated after a median of one year; nutritional condition and quality of life were assessed after a median of two years on a gluten-free diet. The diet was considered strict when there were no signs of dietary transgressions upon the interview. Occasional gluten-free diet was defined as a gluten intake occurring less often than once in the month.

### Small bowel mucosal biopsy

Small bowel mucosal biopsy samples were taken by upper gastrointestinal endoscopy from the distal part of the duodenum. Three samples were paraffin-embedded, processed, stained with hematoxylin-eosin and studied under light microscopy. The villous height/crypt depth ratio (Vh/CrD) was calculated from well orientated biopsy specimens as previously described [9]; a ratio < 2 was considered abnormal and indicative of active celiac disease. The densities of intraepithelial lymphocytes (IELs) were counted from randomly selected surface epithelium and expressed as IELs per 100 epithelial cells [9].

### Celiac serology and HLA

IgA-class tissue transglutaminase antibodies (TGA) were investigated by enzyme-linked immunosorbent assay (Celikey; Phadia, Freiburg, Germany) according to manufacturer's instructions; values ≥5.0 arbitrary units (U) were considered elevated. TGA-positive sera were further analyzed for IgA-class endomysial antibodies (EMA) by an indirect immunofluorescence method using human umbilical cord as substrate; a dilution of 1:5 or more was considered positive [10].

The study patients were genotyped for HLA-DQB1*02, DQB1*0302 and DQA1*05 alleles using the DELFIA Coeliac Disease Hybridization Assay (Perkin-Elmer Life and Analytic Sciences, Wallac Oy, Turku, Finland) according to manufacturer's instructions; DQB1*02 and DQA1*05 corresponding to associated alleles for HLA DQ2 and DQB1*0302 for HLA DQ8.

### Clinical symptoms

The clinical symptoms were classified into three different subgroups: (i) no symptoms, (ii) subtle symptoms with occasionally one or more of the following: abdominal pain, flatulence, belching, loose stools, tiredness, joint pain or oral blisters, and (iii) classical symptoms with constant abdominal complaints, diarrhea or excessive weight loss. Abdominal complaints were additionally evaluated by the Gastrointestinal Symptom Rating Scale (GSRS), which denotes the total score derived from five different gastrointestinal symptoms; diarrhea, indigestion, constipation, abdominal pain and gastro-esophageal reflux; a higher score indicates more severe symptoms [11]. Quality of life was appraised by the Psychological General Well Being (PGWB) questionnaire [12]. This is a 22-item questionnaire including both negative and positive affective states divided into six parts: anxiety, depressed mood, positive well-being, self-control, health and vitality; here a higher score denotes better quality of life. Both questionnaires have been widely employed in celiac disease. A total of 110 subjects served as non-celiac controls for GSRS and PGWB. The controls for GSRS and PGWB were collected form the general population. We asked celiac members of the Finnish celiac society to recruit an individual living in their neighbourhood and not suffering from celiac disease. They had similar age and sex distribution as the study subjects. Celiac disease was not systematically excluded in controls, but the subjects reported no symptoms and had no relatives with celiac disease.

### Assessment of nutritional condition

Body mass index was computed as weight/height^2 ^(kg/m^2^). Blood hemoglobin, erythrocyte folic acid levels, serum iron, ferritin, ionized calcium, phosphate and vitamin A, B-12, D-25 and E concentrations were measured using routine laboratory methods.

### Bone mineral density and history of fractures

Bone mineral density was measured by dual-energy X-ray absorptiometry (GE Medical Systems, LUNAR, UK) in the spine (L1 to L4) and right femoral neck. Values were expressed as standard deviation (SD) scores, which compare individual values to the mean bone mineral density of sex-matched young adults (T score) or of the age- and sex-matched population (Z score). T scores above -1.0 SD represented normal values, scores between -1.0 and -2.4 osteopenia and scores ≤ -2.5 SD osteoporosis. When indicated, bisphosphonate medication together with supplementary calcium and vitamin D were recommended, since it was considered unethical to postpone medical treatment and wait for the effect of a gluten-free diet.

The number of low-energy bone fractures was extracted from the questionnaires of the original GOAL study, equally in the total (n = 2815) series and in study subjects.

### Ethical considerations

The study was accepted by the Ethical Committee of Päijät-Häme Central Hospital, and written informed consent was obtained from all participants. Research is in compliance with the Helsinki Declaration.

### Statistics

Data were given as means with 95% confidence intervals (CI) or medians with lower and upper quartiles and range when appropriate. Chi-square or Fisher's tests were used in cross tabulations Wilcoxon signed or paired rank test to compare changes within the study group. A p value < 0.05 was considered statistically significant. We calculated that, for the statistical power of 0.80 at a significance level of 0.05, 30 subjects would be sufficient. We estimated that the difference of > 0.5 in the Vh/CrD and that of 0.5 in GSRS were clinically significant [13,14].

## Results

### Baseline findings

All 35 new celiac disease patients identified by mass screening consented to participate (Table 1). Celiac disease-related genetic susceptibility markers were found in all. Ten (29%) out of 35 had a family history of celiac disease and 10 (29%) one or several autoimmune diseases. Table 1 displays subjects in whom clinical features would make case-finding by active screening possible. Fourteen were reported to suffer from subtle, one from classical symptoms, and 20 had no symptoms prior to the diagnosis of celiac disease. Blood hemoglobin levels were below reference values in four (13%), serum iron in two (6%), ferritin in nine (26%), vitamin B12 in six (17%), erythrocyte folic acid in 13 (37%), serum phosphate in three (9%) and vitamin E levels in one (3%). Iron or folic acid supplement were given when considered ethically justified. Osteopenia was found in 14 and osteoporosis in eight (altogether in 62%). Eight (23%) out of 35 had a history of low-energy fractures; for comparison, in the whole GOAL study, low-energy fractures were reported in 123 (4%) out of 2815 o subjects, the difference being statistically significant (p < 0.01). None of the new celiac disease patients was under-weight. At baseline, GSRS and PGWB scores did not differ from those in the control series (Table 2), although the GSRS scores were in general higher in celiac patients, indicating more gastrointestinal symptoms.

**Table 1:** Clinical features

Case	Gender Age (y)	tTGA^a^	EMA^b^	Symptoms	Mal-absorption	Diagnostic clues for detecting celiac disease
1	M 52	> 100	1:2000	Subtle		Vitamin B12 deficiency
2	M 52	29.2	1:2000	None	-	Fracture of hand
3	F 52	> 100	1:4000	None	+	Pernicious anemia, hypothyroidism
4	F 52	8.2	1:50	None	+	Blisters in mouth, family history
5	F 52	35.7	1:500	Subtle	-	
6	F 52	> 100	1:2000	Subtle	+	
7	M 53	29.7	1:200	Subtle	-	Sarcoidosis, family history
8	M 53	6.1	1:5	Subtle	-	Family history
9	F 53	6.2	0	Subtle	+	Fracture of vertebra, osteomalacia hypothyroidism
10	M 54	88.3	1:1000	None	+	Fracture of ankle, psoriasis
11	M 54	93.0	1:500	None	+	
12	F 55	> 100	1:500	Subtle	-	Sjögren's syndrome, psoriasis
13	F 55	43.4	1:500	None	-	Osteoporosis
14	M 55	24.5	1:200	Subtle	+	Psoriasis, osteoporosis
15	F 56	58.5	1:200	Classic	-	Family history
16	F 56	18.3	1:50	None	-	Family history
17	F 56	46.6	1:1000	None	-	Type I diabetes mellitus, hypothyroidism
18	M 62	97.4	1:500	None	+	Depression, vitamin B12 deficiency
19	F 63	5.8	1:5	None	**+**	
20	M 63	38.9	1:1000	Subtle	+	Sarcoidosis, osteoporosis
21	F 63	94.1	1:200	None	-	Hypothyroidism
22	F 63	16.5	1:100	None	+	Hyperthyroidism, family history
23	F 64	71.2	1:500	None	+	Fracture of ribs and sternum, osteoporosis
24	M 64	5.8	1:5	None	+	Fracture of wrist
25	F 64	10.5	1:5	None	-	
26	F 65	> 100	1:500	Subtle	+	Osteoporosis
27	F 65	64.3	1:200	Subtle	-	Hypothyroidism, family history
28	F 66	79.8	1:500	Subtle	**+**	Hypothyroidism, family history
29	M 72	> 100	1:200	Subtle	+	Fracture of foot, family history
30	M 73	27.4	1:50	Subtle	+	
31	M 73	20.2	1:200	None	+	Fracture of ribs, osteoporosis blisters in mouth
32	M 75	6.0	1:5	None	+	Osteoporosis
33	F 76	7.4	1:5	None	-	Osteoporosis
34	M 76	5.4	1:5	None	+	Fracture of ribs, family history
35	F 76	48.3	1:500	None	-	Stomach cancer

Clinical features of screen-detected celiac disease patients having small bowel mucosal villous atrophy at the time of diagnosis.

^A^ Serum IgA-class tissue transglutaminase antibodies, values > 5.0 U/l abnormal

^B^ Serum IgA-class endomysial antibodies, titers 1:≥5 abnormal

^C^ Sarcoidosis treated with immunosuppressive medication

**Table 2:** Symptoms and quality of life

	Screen-detected celiac patients				
	At the diagnosis After gluten-free diet		p-value^a^	Non-celiac controls	p-value ^b ^
GSRS ^c^					
Diarrhea	2.1 (1.7-2.6)	1.5 (1.2-1.9)	0.009	1.6 (1.5-1.8)	0.091
Indigestion	2.8 (2.4-3.2)	2.0 (1.6-2.3)	< 0.001	2.4 (2.0-2.7)	0.117
Constipation	2.1 (1.7-2.5)	1.8 (1.5-2.1)	0.343	2.0 (1.6-2.3)	0.813
Abdominal pain	2.1 (1.7-2.4)	1.7 (1.4-2.0)	0.020	1.8 (1.5-2.1)	0.203
Reflux	1.8 (1.4-2.2)	1.4 (1.2-1.7)	0.040	1.5 (1.8-1.2)	0.161
Total score	2.2 (1.9-2.5)	1.7 (1.5-2.0)	0.001	1.9 (1.7-2.1)	0.133
PGWB^d^					
Anxiety	23 (21-24)	24 (22-25)	0.256	25 (24-26)	0.063
Depression	16 (15-17)	16 (15-17)	0.429	16 (16-17)	0.713
Well-being	16 (15-17)	17 (16-18)	0.011	17 (16-17)	0.364
Self-control	15 (14-16)	15 (14-16)	0.942	15 (15-16)	0.593
General health	13 (12-14)	12 (11-14)	0.243	14 (14-15)	0.030
Vitality	17 (16-18)	18 (16-19)	0.100	19 (18-19)	0.110
Total score	100 (93-106)	102 (95-109)	0.620	106 (104-108)	0.355

Gastrointestinal symptom score (GSRS) and psychological general well-being (PGWB) in older celiac disease patients before and after dietary treatment, compared to non-celiac controls

^a ^Comparison between celiac patients at the diagnosis and after gluten-free diet

^b ^Comparison between celiac patients at the diagnosis and non-celiac controls

^c ^Gastrointestinal symptom rating scale; higher scores indicate more gastro-intestinal symptoms

^d ^Psychological general well-being; higher scores indicate better quality of life

### Gluten-free dietary treatment

Thirty-two (91%) of the 35 screen-detected older celiac disease patients consented to start a gluten-free diet. Twenty-seven maintained a strict diet, and five had occasional transgressions less often than once in the month; three patients did not commence gluten-free diet.

**Figure 1:**
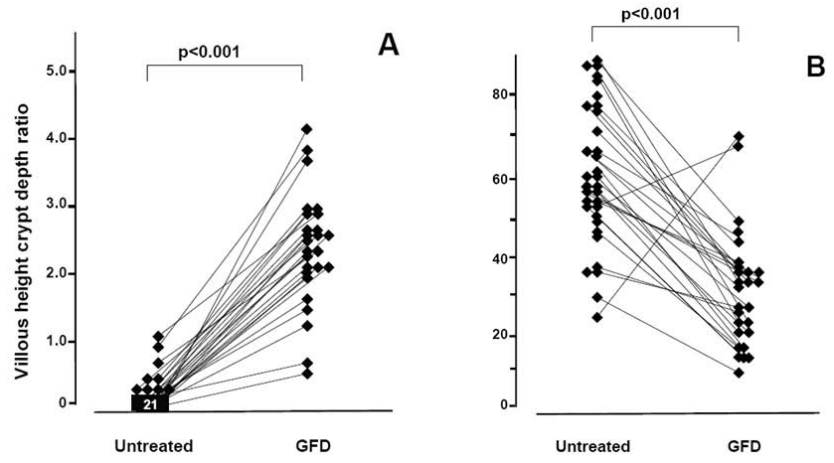
**Small bowel mucosa before and after gluten-free diet**. The villous height crypt depth ratio (A) and the densities of intraepithelial lymphocytes/100 enterocytes (B) at the time of diagnosis and after one year on a gluten free diet (GFD) in older celiac disease patients detected by mass-screening.

After one year on the diet, small bowel mucosal villous morphology improved and densities of IELs decreased statistically significantly in the 26 who agreed to undergo the follow-up biopsy (Figure 1). In parallel, serum TGA levels normalized.

Clinical symptoms resolved in 14 out of the 15 who reported symptoms at the time of diagnosis. One with initially subtle abdominal complaints developed diarrhea despite small bowel mucosal normalization on a strict gluten-free diet; colonoscopy showed no abnormal findings or inflammation. In accordance with the clinical history, alleviation in gastrointestinal symptoms was observed both by GSRS total score and by subscores, and was statistically significant, except in the case of constipation (Table 2).

Mean serum ferritin and vitamin B_12_, D and E values improved significantly in those 32 who started the gluten-free diet (Table 3). By contrast, mean serum vitamin A and ionized calcium values decreased after commencement of diet, albeit remaining within normal reference range. There was also a small albeit statistically significant decrease in blood hemoglobin levels in females. To verify that the beneficial effects were due to gluten-free diet, we analyzed separately patients who did not receive any additional medical iron or vitamin substitution. The beneficial effect remained significant in serum iron (12 excluded, p = 0.042), serum ferritin (12, p > 0.001), vitamin B_12 _(14, p = 0.039), and vitamin D (21, p = 0.006).

**Table 3:** Malabsorption

	Reference values	At the diagnosis		After gluten-free diet		p-value
		Mean	Range	Mean	Range	
Blood hemoglobin	M: 13.4-16.7 g/dl	14.1	11.6-16.4	14.3	11.9-16.0	0.225
	F: 11.7-15.5 g/dl	13.8	12.0-15.6	13.3	10.7-16.1	0.019
Serum iron	10-34 μmol/l	17	5-28	19	9-40	0.280
Serum ferritin	M: 20-275 μg/l	46	5-177	120	7-351	0.004
	F: 7-205 μg/l	33	5-78	83	7-351	0.001
Serum vitamin B12	150-740 pmol/l	275	110-542	355	142-739	0.018
Erythrocyte folic acid	180-845 nmol/l	290	88-662	403	108-1292	0.058
Serum vitamin D25	22-103 nmol/l	45	15-77	64	29-97	< 0.001
Plasma phosphorus	0.71-1.53 mmol/l	0.94	0.59-1.19	0.93	0.72-1.24	0.382
Serum vitamin A	1.0-3.0 μmol/l	2.2	1.1-3.3	2.0	1.0-2.5	0.025
Serum vitamin E	12-48 μmol/l	30	10-42	35	25-65	0.005
Serum ionized calcium	1.15-1.30 mmol/l	1.23	1.15-1.33	1.22	1.15-1.32	0.049

Malabsorption parameters at the diagnosis and after gluten-free diet in 32 screen-detected older celiac disease patients. Some of the patients received also vitamin or iron supplements, see the text.

The mean femoral Z-score improved significantly when patients had adhered to the gluten-free diet for two years; in the lumbar spine the improvement did not reach statistical significance (Table 4). There were no significant changes in T-scores. In subjects with osteopenia or osteoporosis, the mean T-score increased in lumbar spine from -2.0 (95%CI -2.3 to -1.7) to -1.7 (95%CI -2.1 to-1.3), and in femoral neck from -2.2 (95%CI -2.5 to -1.9) to -2.1 (95%CI -2.4 to -1.8) (p-values 0.03 and 0.20, respectively). Bisphosphonates were prescribed for three patients with osteoporosis or osteopenia. When these patients were excluded from the analysis, the increase in lumbar spine T-score remained still statistically significant (p = 0.021). The diet had no obvious effect on BMI. Quality of life measured by PGWB did not change during the follow-up; apart from improvement in the well-being score (Table 2). The three patients who declined the diet initially reported no symptoms, but developed minor abdominal complaints after the follow-up; in two osteopenia and low folic acid levels remained.

**Table 4:** Bone density and body mass index

	At the diagnosis		After gluten-free diet		
	Mean; 95% confidence intervals		Mean; 95% confidence intervals		
Lumbar spine Z-score (SD)	0.6	0.0 - 1.1	0.9	0.1 - 1.5	0.060
Femoral Z-score (SD)	-0.1	-0.5 - 0.2	0.1	-0.2 - 0.6	0.009
					
Lumbar spine T-score (SD)	-0.7	-1.2 - -0.1	-0.4	-1.2 - 0.1	0.241
Femoral T-score (SD)	-1.1	-1.5 - -0.6	-1.1	-1.5 - -0.5	0.670
					
Body mass index (kg/m^2^)	25.9	24.6-27.1	25.3	24.1-26.5	0.139

Bone mass density (T and Z -score) and body mass index (mean with 95% confidence intervals (CI)) at diagnosis and after gluten-free diet in 32 screen-detected older celiac disease patients

## Discussion

An increasing number of patients with celiac disease will be diagnosed among the older people [1-3]. The correct diagnosis may have been missed even when the patients had contacted their physicians for many years due to unexplained symptoms or abnormalities in blood tests [3]. Altogether, older patients may have more symptoms than younger ones [15], and may have an increased risk of malabsorption or enteropathy-associated T-cell lymphoma[16]. Anemia, iron, vitamin B12, folic acid, and calcium deficiency have been the major malnutrition findings in older celiac disease patients [3,16]. It would thus, appear desirable to detect the disease as early as possible.

On the other hand, it is of crucial importance to know what the overall implications of the diagnosis are in older people. Mortality has not increased among older undiagnosed celiac disease patients [6] and the effect of the diagnosis on well-being has not been investigated. In this prospective follow-up study we evaluated the consequences of dietary treatment in a definite celiac disease patient series obtained by population-based mass screening in the older [17]. Since neither celiac disease nor any abdominal disease was the target of the original GOAL project, there was no selection bias towards individuals suffering from gastrointestinal symptoms. Of note, the rate of detection of celiac disease in Finland is relatively high, and in the present series, 0.9% of individuals already had the diagnosis of celiac disease established before the screening program [17]. This notwithstanding, even here the majority of older celiac disease patients would have remained undiagnosed without active screening or case finding.

In these screen-detected patients an improvement in GSRS was evident under dietary treatment, both in total score and in virtually in all subscores, displaying an alleviation in gastrointestinal symptoms. The effect of the treatment on quality of life (PGWB) was not so evident, but it is of note that the diet did not worsen it. A comparable finding was obtained in our recent study where celiac patients detected by screening at risk groups were investigated [18]. An improvement in laboratory values was seen almost invariably. This was most evident in serum mean ferritin indicating the presence of subclinical iron deficiency, as the serum iron levels remained within normal range. A low ferritin level was similarly observed in a series from Godfrey and colleagues [6]. Apart from gluten-free diet, iron or vitamin supplementation was given to some of our patients, but the beneficial effect was evident also in those subjects, who did not receive any substitution. There was a slight but statistically significant decrease in blood hemoglobin levels in females (Table 3), but none of the patients suffered from severe anemia. A regular follow-up of hemoglobin values is in any case indicated in celiac patients.

A risk of low bone mineral density is possible in screen-detected apparently asymptomatic celiac disease patients [5]. In the present study, Z-scores, reflecting the values in the age- and sex-matched population, were within reference levels at baseline, but a significant improvement was observed on a gluten-free diet. As Z-score reference values usually decrease with age, we concluded that this process was slowed down by dietary treatment. Though no improvement was observed in T-scores in the total series, such an effect was seen in subjects with osteoporosis or osteopenia, even when subjects treated with bisphosphonates were excluded. The analysis would have been impossible if also subjects receiving vitamin D or calcium substitution were excluded. On the other hand, in the randomized study carried out by Mautalen et al. [19], strict gluten-free diet promoted a significant increase in bone mineral density, but calcium and vitamin D supplementation did not provide additional benefit. We will further point out, that the medical management for bone disease of malabsorption would not have been possible without our active screening. There was a small but significant decrease in serum calcium levels. This may be due to ongoing bone restoration, which implies that calcium substitution is indicated after the commencement of a gluten-free diet. Our results further suggest that low-energy fractures may be a risk in untreated celiac disease. Larger prospective studies are however needed to confirm this finding.

Compliance with a gluten-free diet does not seem to be a problem in older patients with celiac disease, as compliance rates have been more than 90% [3]. Accordingly, the histological or serological recovery in the 32 patients adhering to a gluten-free was virtually complete. However, our results cannot directly be applied in every country, as the availability of gluten-fee diet may not be as good as in Finland. Another limitation of the study was that we did not have laboratory or bone mineral density values for the control group. Nevertheless, we emphasize that a favorable outcome can be achieved by screening older population for celiac disease.

None of our celiac disease patients suffered from severe malabsorption syndrome, and they did not have refractory sprue or any other severe complications. Ten (29%) of these 35 screen-detected celiac patients had had relatives with celiac disease and 10 autoimmune diseases, which should both alert to celiac disease. Katz and associates [20] concluded that symptoms do not predict who will have celiac disease, making case-finding ineffective, and they therefore suggested that general population screening may be needed to find the disorder. To the contrary, we believe that in this older population case finding will be effective as long as symptoms and risk-groups are taken into account. Altogether, 29 out of 35 of our celiac patients would have been detected without serologic mass-screening if family history, bone fractures or concomitant diseases (Table 1) had alerted the physicians (in patient 35 routine duodenal biopsy would have detected celiac disease). We therefore recommend screening in groups, where the costs are lower than in mass-screening, and as shown here, the patients benefit from dietary treatment.

## Conclusions

Screen-detected, apparently asymptomatic older celiac disease patients may suffer from subclinical malabsorption, gastrointestinal symptoms or bone disease, which are alleviated during gluten-free dietary treatment. No deterioration in quality of life was seen in our series, and dietary compliance was excellent. Despite this, we consider that there is still insufficient evidence to advocate mass screening, until the costs and benefits of the approach have been thoroughly evaluated. Instead, as the majority of patients had a family history or associated conditions known to occur with celiac disease, we recommend active case finding in older people belonging to at-risk groups.

## Competing interests

The authors declare that they have no competing interests.

## Authors' contributions

AV, KK, LL, MM and PC conceived the study participated in the patient enrolment, data collection and study design: IK, ML and HP participated in the data collection and study design. RV and KL carried out the immunological studies and participated in the study design. All authors read and approved the final manuscript.

## Pre-publication history

The pre-publication history for this paper can be accessed here:

http://www.biomedcentral.com/1471-230X/11/136/prepub
